# Upscaling Genotyping by Amplicon Sequencing With GBAS‐GUI

**DOI:** 10.1111/1755-0998.70198

**Published:** 2026-09-04

**Authors:** Sebastian Sonnenberg, Thapasya Vijayan, Christina Rupprecht, Yoko Philipina Krenn, Melissa Gruber, Hannah Dorfer, Gerald Kwikiriza, Harald Meimberg, Manuel Curto

**Affiliations:** ^1^ Institute of Integrative Nature Conservation Research, Department of Ecosystem Management Climate and Biodiversity, BOKU University Vienna Austria; ^2^ Aquaculture Research and Development Centre Kajjansi (ARDC), National Fisheries Resources Research Institute (NaFIRRI), National Agricultural Research Organization (NARO) Kampala Uganda; ^3^ CIBIO, Centro de Investigação Em Biodiversidade e Recursos Genéticos, InBIO Laboratório Associado, Campus de Vairão, Universidade do Porto Vairão Portugal; ^4^ BIOPOLIS Program in Genomics, Biodiversity and Land Planning, CIBIO, Campus de Vairão Vairão Portugal

**Keywords:** amplicon sequencing, EPIC markers, gene‐duplication, genetic monitoring, graphical user interface, microsatellites, nuclear markers, polymorphism information content, SSR‐seq

## Abstract

Genotyping by amplicon sequencing (GBAS) is a relatively low‐cost approach for generating genotypic data compared with established genomic methods, making it highly scalable and particularly suitable for large‐scale genetic monitoring projects. However, most existing analytical pipelines are either marker‐specific, insufficiently scalable, or lacking efficient data management systems for the long‐term integration of genotypic information, limiting the full potential of GBAS. Here, we address this gap by introducing GBAS‐GUI (https://github.com/sonnenbe‐dot/GBAS‐GUI), a pipeline capable of generating GBAS‐based genotypic data for a wide variety of loci at scale. GBAS‐GUI integrates a graphical user interface with multiple checkpoints to improve accessibility and robustness. It implements multiprocessing architecture and a relational database that links genotypic data with associated sample metadata to enhance scalability and data management. The pipeline further enables marker screening through automated calculation of polymorphism information content (PIC) and implements a strategy to recover homologous genotypic information from paralogous loci with non‐overlapping amplicon length ranges. Using multiple empirical datasets, we demonstrate substantial improvements in processing speed, database management and handling artefacts related to co‐amplification of unspecific regions and duplicates of the same genomic region. We further show that incorporating the full sequence information captured by an amplicon increases marker information content beyond what is achievable with length‐based genotyping alone and expands the analytical versatility of GBAS. Overall, GBAS‐GUI provides a robust, scalable and versatile framework that unlocks the potential of GBAS for large‐scale population genetic and phylogeographic studies.

## 1 Introduction

Genotyping‐by‐sequencing (GBS) involves the use of high‐throughput sequencing (HTS) technologies to produce large‐scale genotypic data. Although GBS is better known through methods like RAD‐sequencing, which allow researchers to obtain genome‐wide genotypic information for non‐model species (Baird et al. 2008), it also includes amplicon sequencing (Tibihika et al. 2020). This approach involves sequencing Polymerase Chain Reaction (PCR) products targeting specific loci, a strategy known as genotyping by amplicon sequencing (GBAS; Eriksson et al. 2020; Vartia et al. 2016). One of the first examples was the Genotyping‐in‐Thousands approach, which genotyped 192 SNPs in 2068 individuals of steelhead trout (Campbell et al. 2015).

By combining GBAS with established marker systems (Curto et al. 2025; De Barba et al. 2017; Stuglik et al. 2011) it is possible to make the genotyping process more cost‐ and labour‐efficient (Yang et al. 2019), scalable (Pawula et al. 2023; Pimentel et al. 2018) and replicable, while increasing its information content (Curto et al. 2019; Šarhanová et al. 2018). These advantages arise from several properties specific to amplicon sequencing. The combination of multiplex PCR with HTS enables large‐scale genotyping of dozens of loci at relatively low cost (De Barba et al. 2017; Vartia et al. 2016). The simple and standardized format of sequencing data increases the reproducibility and allows for automation of genotyping approaches (Curto et al. 2019; Tibihika et al. 2019). Furthermore, when used to genotype length‐based marker systems such as microsatellites, it increases the number of alleles detected and overcomes issues of size homoplasy and artefacts inherent to capillary electrophoresis (Curto et al. 2019; Neophytou et al. 2018; Šarhanová et al. 2018; Tibihika et al. 2019). In this way, GBAS helps keep classical markers relevant in the genomic era, particularly given their applicability in large‐scale surveys such as genetic monitoring programs (Schwartz et al. 2007), forensic applications including fingerprinting and parentage analysis (Hodel et al. 2016; Vieira et al. 2016).

The analysis of amplicon sequencing data is not trivial, as both PCR and sequencing steps introduce errors that may be mistakenly interpreted as true genetic variation (Pimentel et al. 2018). A wide range of pipelines and analytical approaches have been developed to address this challenge. The main bioinformatic developments for amplicon sequencing have focused on metabarcoding applications (Dong et al. 2025; Luo et al. 2026), where the primary objective is to recover sequence variants and/or taxonomic composition from mixed‐community samples, rather than to infer individual multilocus genotypes. Consequently, these approaches do not directly address several challenges associated with GBAS data. Most were designed specifically for microsatellites and do not account for other types of variation beyond simple sequence repeat (SSR) changes (e.g., Ganschow et al. 2018; Huo et al. 2021; Liu et al. 2024). To our knowledge, only a few pipelines can integrate all the information captured by a single amplicon (e.g., Barbian et al. 2018; King et al. 2017; Sebastian et al. 2016). Many of these focus primarily on SNP genotyping or haplotyping and therefore do not account for artefacts specific to markers characterized by high length polymorphism like microsatellites (e.g., Campbell et al. 2015; Sebastian et al. 2016; Stuglik et al. 2011). Others, like HipSTR (Willems et al. 2017), were developed for shotgun sequencing data and require mapping to a reference genome, which is not always available. Furthermore, there are methods, like the approach described by Šarhanová et al. (2018), that require extensive manual curation, limiting their scalability. Although some tools automatically use complete amplicon sequences to define alleles, fully automated implementations may still result in considerable error rates (Salado et al. 2021), thus, visual inspection remains necessary for reliable genotype calling. Finally, to our knowledge no analytical approach explicitly addresses the duplicated nature of eukaryotic genomes. Polyploidy, gene duplication and pseudogenization, together with the co‐amplification of paralogous or nonspecific PCR products, can lead to erroneous genotype calls, particularly when analyses assume diploidy. Existing approaches that attempt to disentangle such signals are typically designed either for polyploid genotyping or for the analysis of gene families and are therefore not easily applicable across a broad range of genetic markers (Cui et al. 2022; Stuglik et al. 2011).

In the present study, we introduce GBAS‐GUI that addresses these challenges. This method calls genotypes in two steps. First, it identifies alleles based on amplicon length, mimicking traditional capillary electrophoresis. Second, it defines haplotypes within each length class by incorporating SNP information. In the first step, the allele call is guided by expected length–depth ratio distributions. The pipeline also provides graphical support for manual validation and correction of genotype calls. Allele identification based on full sequence information is then performed by detecting potential SNPs while ignoring other sources of within‐length variation. Together, these steps progressively reduce data complexity and exclude potential artefacts. This genotype calling strategy has already been implemented in the set of Python scripts that users must execute sequentially (https://github.com/mcurto/SSR‐GBS‐pipeline) first published by Curto et al. (2019) and Tibihika et al. (2019). Since then they have proven effective in generating genotypic data for a wide range of marker types as well (Agoe et al. 2026; Curto et al. 2025; Kendlbacher et al. 2025; Vijayan et al. 2026). GBAS‐GUI formalizes this genotyping approach into a single program wrapped in a graphical user interface (GUI) making it more accessible and user‐friendly, while incorporating multiple checkpoints to ensure that user‐defined parameters are correctly set and runtime errors are prevented. GBAS‐GUI uses a parallelization framework making GBAS more scalable to larger data volumes characteristics to genetic monitoring projects. In addition, it incorporates a relational local database that integrates genotypic and allelic data with associated metadata such as species identity and geographic origin, facilitating data organization and data retrieval. GBAS‐GUI also implements automated calculation of polymorphism information content (PIC) per marker, enabling an initial assessment of marker informativeness. Finally, we introduce a strategy that defines multiple amplicon‐length intervals per locus to recover genotypes from the co‐amplification of duplicated genomic regions or nonspecific PCR products. Therefore, GBAS‐GUI provides a robust and scalable framework for reliable genotyping across diverse amplicon marker types while improving data management and analytical reproducibility.

## 2 Material and Methods

### 2.1 Pipeline Description

#### 2.1.1 Allele Call Algorithm

**FIGURE 1:**
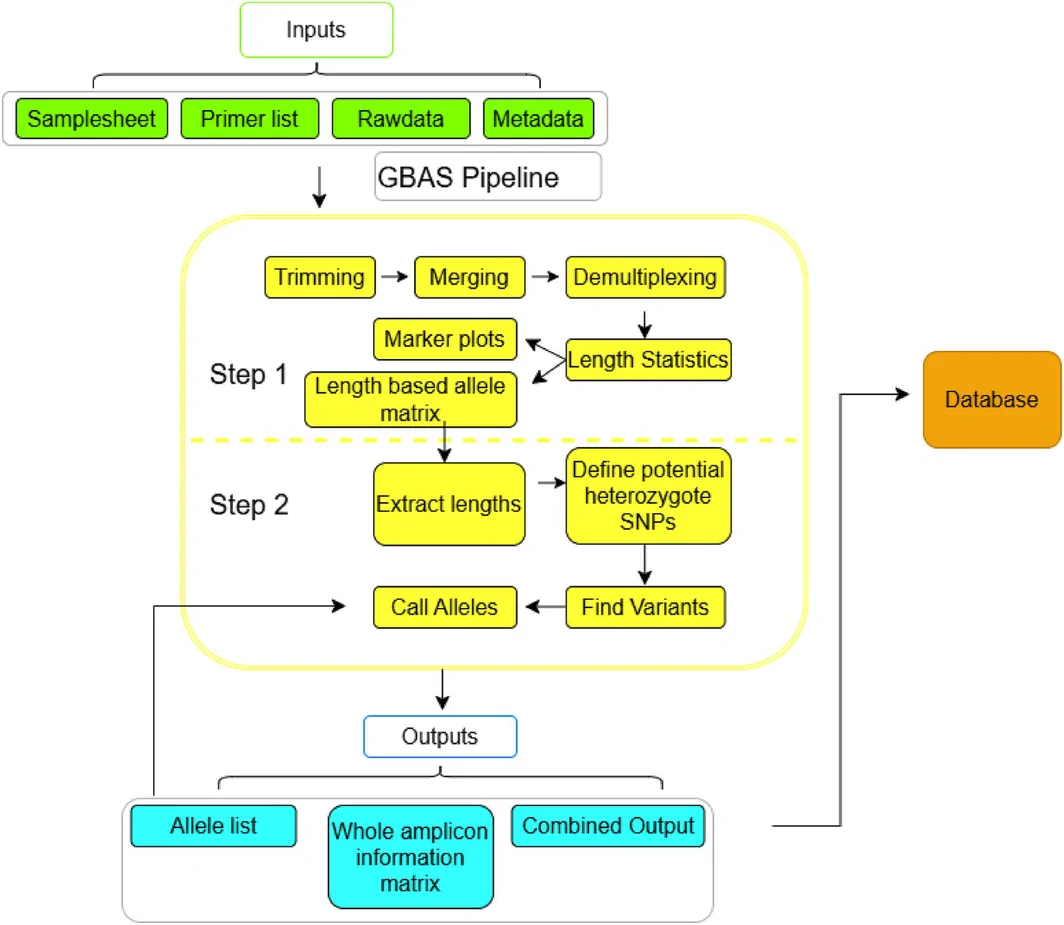
Workflow behind the GBAS‐GUI pipeline. Green boxes correspond to input files, yellow boxes to the processes of the two main steps of the pipeline, while blue boxes to the main output files. The relational database is coloured orange.

GBAS‐GUI produces genotype information in two sequential steps. Here we provide a brief overview (Figure 1).

Step 1: Raw paired‐end reads are quality trimmed and filtered, and paired reads are merged using the third‐party programs *Trimmomatic* (Bolger et al. 2014) and *USEARCH* (Edgar 2010). The resulting merged reads are demultiplexed based on primer sequences. For each sample and locus, amplicon‐length distributions are computed from read counts. Peaks in these distributions are used to define length classes consistent with the expected ploidy level (diploid or haploid), which are interpreted as candidate length‐based alleles following (Curto et al. 2019). These are stored in a codominant matrix. Amplicon‐length histograms generated by the pipeline allow visual inspection of length distributions, helping to identify potential problematic markers that can either be excluded or rescued as described in Section 2.1.6.

Step 2: Sequence‐based allele call: Only sequences belonging to the allele‐length class retained in Step 1 are kept, reducing both sequence noise and consequently its complexity. By comparing sequences of the same length within each sample and locus, potential heterozygote SNPs site can be identified without sequence alignment. This is achieved by calculating frequencies at each position across all reads. Positions where no nucleotide exceeds a user‐defined threshold are treated as candidate heterozygous SNP sites. The pipeline then evaluates haplotypes formed by combinations of nucleotides at these positions that are supported by the read data, while variation at other positions is assumed to result from sequencing errors and is ignored. Final genotypes are determined based on the frequency of the supported haplotype variants. Ambiguity in genotype definition—for example, when the number of equally abundant variants exceeds the expected ploidy level—results in a missing genotype call. These are then compiled into a codominant genotype matrix together with an allele list. The allele list serves as a reference key to ensure allele comparability across analyses when new data are incorporated at a later stage. In addition, FASTA files containing the final sequence variants are also provided in one of the intermediate outputs.

To run the pipeline, in addition to paired FASTQ files generated with Illumina sequencing, the user needs to provide a sample sheet containing corresponding sequence file names and a primer file listing all markers and corresponding primer sequences. A file containing metadata per sample can additionally be provided. More information regarding the format of the inputs can be found on the official documentation at https://github.com/sonnenbe‐dot/GBAS‐GUI.

#### 2.1.2 Graphical Interface

A graphical user interface (GUI) was developed to allow running the GBAS‐pipeline in a straightforward way, accessible to users regardless of their bioinformatics background. The GUI application was written in python using the custom UI‐library CustomTKinter (https://github.com/TomSchimansky/CustomTkinter).

**FIGURE 2:**
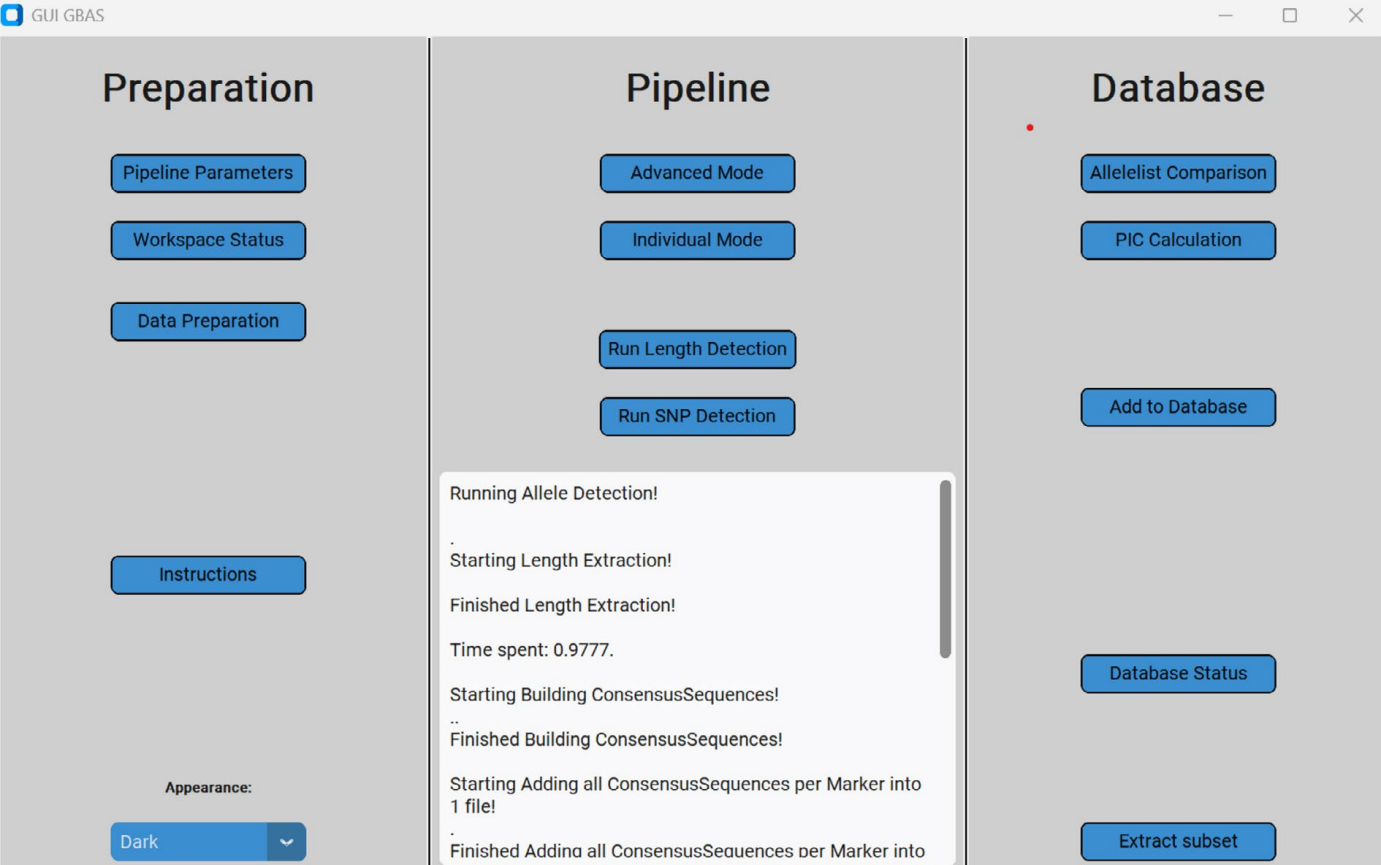
Main window of the GUI organized into three sections corresponding to the main stages of running the pipeline: Preparation, Pipeline and Database.

The GUI is organized into three sections that correspond to three main stages of running the pipeline (Figure 2). The ‘Preparation’ section contains functions for setting up all inputs, checking if they are correctly formatted and valid, as well as an additional function of importing samples metadata based on the specific fields of the sample sheet file. The ‘Pipeline’ section handles the execution of the pipeline in standard sequential mode (Step 1: Length detection; Step 2: SNP Detection), or parts of the pipeline. This can either be done by running combinations of processes (Advanced Mode) or by running individual processes (Individual Mode). The ‘Database’ section allows the user to store pipeline outputs into a local SQLite database and to extract subsets based on user‐defined parameters. This section also enables the calculation of PIC (Polymorphism Information Content) values. This is important for a first evaluation of marker quality. Furthermore, allele lists generated across different batch runs can be compared to quickly identify differences in markers and alleles.

#### 2.1.3 Database

GBAS has a high applicability in large scale studies, involving large sample sets and multiple marker panels that may need to be sequenced in multiple runs. This can become increasingly difficult to manage in terms of storage and data retrieval as more data are added to the project. Thus, it is of utmost importance to have access to a standardized storing procedure across all runs and marker sets used in a GBAS study. In GBAS‐GUI we address this need by implementing a relational database structure to allow for a centralized storing system of multiple projects from different marker systems.

**FIGURE 3:**
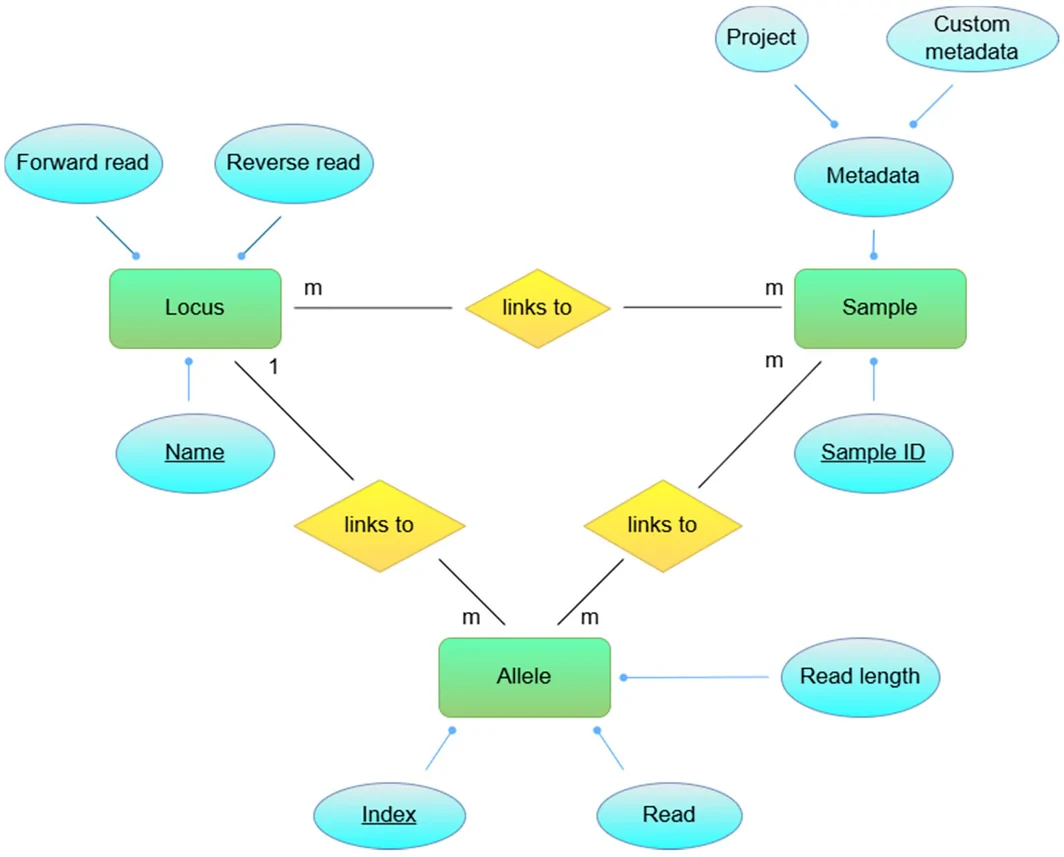
Entity‐Relationship‐Diagram (ER) representing the local database. Rectangles denote the main tables storing information on markers (Locus), samples (Sample) and identified alleles (Allele). Ovals represent attributes or sub attributes of composite attributes. Diamonds indicate relationships linking the main tables and defining how records in different tables are associated. Cardinality between tables is indicated by ‘1’ (one) and ‘m’ (many).

Relational database management systems allow for efficient storage as well as quick retrieval of data subsets through SQL (Standard Query Language). The structure of the relational database implemented in GBAS‐GUI is illustrated in Figure 3 in the form of an Entity‐Relationship‐Diagram (ER). The basic elements of an ER diagram are entities (rectangles), attributes (ovals) and relationships between entities (diamonds). In a relational database structure, data is stored in tables, with each table consisting of rows and columns. Each entity in the diagram is translated into its own table, and each attribute connected to the entity represents a unique column in that table. Relationships are translated depending on their cardinalities, indicated by 1 or m near the entities. Relationships with m:m cardinalities are translated into new tables. Each entity has an underlined attribute that represents its primary key, the attribute that uniquely identifies each row in the table representation.

The database consists of five tables: three main tables (Sample, Locus and Allele) and two link tables (Allele‐Sample and Locus‐Sample), which establish relationships between sample, locus and allele information. Each main table can have several attributes, some of which are unique identifiers for each row. All attributes are fixed, except for a user‐defined metadata field (Custom metadata). The tables are connected by having either a single attribute in common between them or multiple ones.

Each main table stores information derived directly from the users input files (e.g., sample metadata and primer information) or generated during the execution of the pipeline (e.g., amplicon length associated with each allele). The database currently supports up to five metadata fields per sample, of which two are mandatory: sample name and project identifier. Samples lacking project identifiers are excluded from database storage to ensure consistency and traceability of records.

**FIGURE 4:**
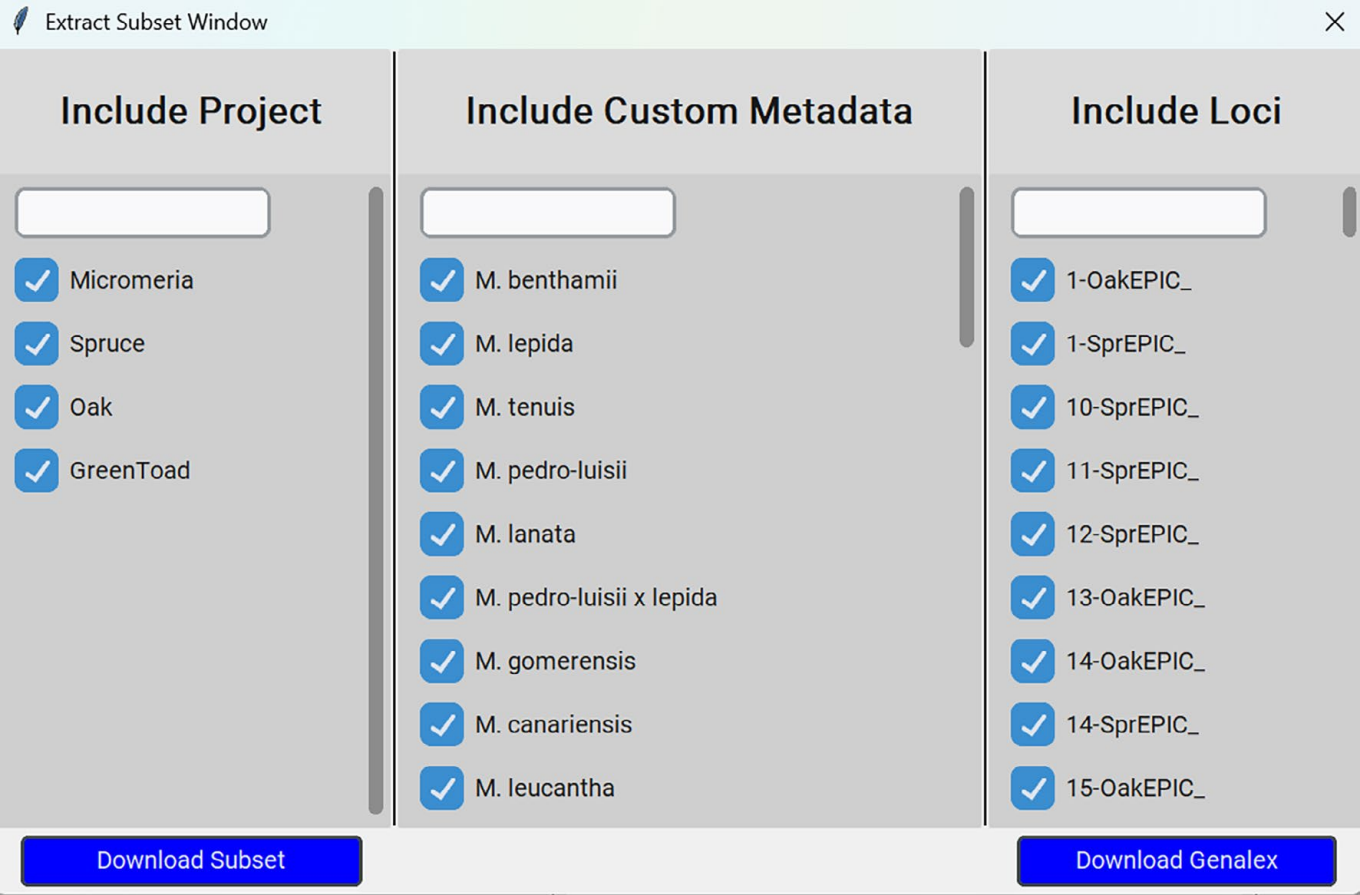
Sub‐window for querying the database. While the variables ‘Project’ (left) and ‘Loci’ (right) are fixed, the third variable Custom Metadata, represented in the middle column, is user defined and defined by the third column in the metadata input file.

Database querying can be done using project identifier, marker name and an additional user‐defined metadata field (Figure 4) which is always the third column in the meta‐data file. This can be any variable relevant for data management such as species or locality. Query results can be exported as codominant genotype matrices, as well as JSON files containing the corresponding subset of the database.

#### 2.1.4 Parallelization

We improved pipeline efficiency by implementing a multiprocessing framework that allows computationally intensive processes to be executed in parallel, significantly reducing computation time. This was achieved by using a Pool from Python's multiprocessing module to distribute tasks across CPU cores. We used the starmap‐async method to enable more dynamic parallelization and ensure efficient use of processing resources. This is particularly important when processing large read files, as it prevents individual processors from being tied to specific files for the entire duration of the run.

Parallelization was applied to the following steps, each of which processes multiple files independently and therefore does not require data sharing across cores: quality trimming, merging, demultiplexing and consensus sequence construction. The maximum number of cores used is defined by the user. By default, this number is set to the maximum number of cores available on the current system minus one to accommodate other background processes.

#### 2.1.5 Additional Output Files

In addition to the matrix and allele list outputs, the GBAS‐GUI produces a combined output of both in JSON format. JSON remains a popular lightweight data format representing a hierarchical structure with easy parsing integration in programming languages. Furthermore, sequence data from the allele list is output in FASTA format.

#### 2.1.6 Rescuing Genotypes From Duplicated Loci or Loci Impacted by Unspecific Products

GBAS‐GUI can disentangle allelic variation from markers that amplify duplicated genomic regions (e.g., due to gene duplication) as well as markers affected by high levels of nonspecific co‐amplification. This approach is effective when different amplified copies or non‐specific products, produce amplicons with non‐overlapping length ranges.

In such cases, users can define specific amplicon‐length intervals corresponding to each copy or amplification product. Length‐based genotypes are then called independently within each interval. Genotypes derived from different intervals are subsequently treated as independent markers by downstream components of the pipeline. These intervals can also be used to exclude nonspecific amplification products from genotyping by omitting their corresponding length ranges from the analysis.

**FIGURE 5:**
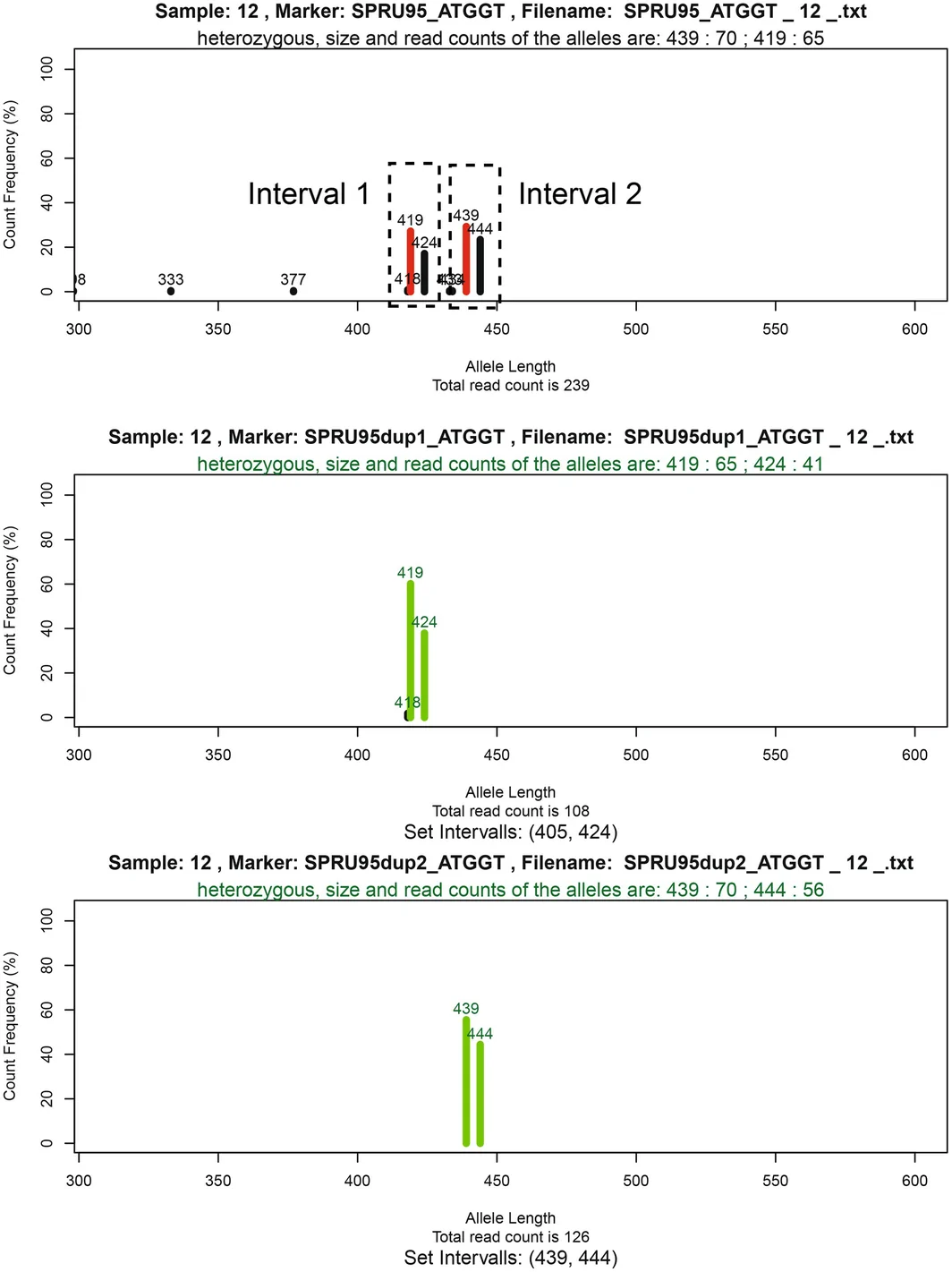
Amplicon length distribution histograms showcasing the example of a duplicated locus with copies with non‐overlapping amplicon lengths (top). Two amplicon length intervals were defined to genotype both copies in an independent way (middle and bottom plots).

The identification of appropriate length boundaries requires prior knowledge of locus duplication or co‐amplification status. This information can be obtained by performing an initial run without specifying any length intervals. The resulting amplicon length histograms can then be inspected for patterns consistent with duplication, such as clearly separated or multimodal length distributions indicative of multiple copies (Figure 5). Once suitable boundaries are defined, the pipeline can be rerun with these parameters to recover copy‐specific genotypes.

#### 2.1.7 PIC Calculation

GBAS‐GUI implements a module to calculate the Polymorphism Information Content (PIC), a standard measure of marker informativeness useful for preliminary assessment of genotype data. PIC is computed for both amplicon‐length–based genotypes (AL) and sequence‐based genotypes derived from whole amplicon information (WAI). The pipeline also reports the difference between these values (PIC_WAI_—PIC_AL_), allowing users to evaluate the increase in informativeness gained by incorporating sequence‐level variation.

PIC estimates and their differences are visualized as automatically generated barplots and boxplots. The results are stored both in a JSON file summarizing the full database and in separate CSV files for each project.

### 2.2 Key Examples

To evaluate the performance and accuracy of GBAS‐GUI, we tested the pipeline on four empirical datasets. These datasets were selected to assess (i) improvements in computational efficiency achieved through parallelization, (ii) increases in marker informativeness when using whole amplicon information (WAI) instead of amplicon‐length (AL) genotypes, (iii) the ability to correctly handle duplicated loci, and (iv) the accuracy of sequence‐based allele calls when compared with Sanger sequencing.

The four datasets comprise either microsatellites, nuclear genes or a combination of both:

- Green toad: Genotyping data for 48 nuclear genes and 45 microsatellite markers from 288 individuals of *Bufotes viridis* . This dataset represents a typical population‐genetics sampling scheme with a large number of individuals.
- Oak: Genotyping data for 41 nuclear genes and 80 microsatellite markers from 120 individuals of *Quercus* spp. Another typical population genetics sampling scheme but with a higher number of markers than the Green Toad.
- Spruce: Genotyping data for 52 nuclear genes and 95 microsatellite markers for 102 individuals of *Picea abies* . As *P. abies* has a highly repetitive genome, this dataset is well‐suited for testing the efficiency of the pipeline's duplication‐handling strategy. Furthermore, it illustrates the application of GBAS with large marker panels.
- Micromeria: Genotyping data from Curto et al. (2025) composed of 283 individuals and 24 nuclear genes. A subset of three nuclear genes and 21 individuals was used to assess sequence‐level accuracy through comparison with independent Sanger data.

Information regarding sample origin, data availability, and markers used can be found in Supporting Information (Tables S1 and S2). The green toad samples consisted of mouth swabs that were processed and analyzed exactly as described by Kendlbacher et al. (2025) while the Oak and Spruce datasets were as described in Vijayan et al. (2026). The *Micromeria* data was taken directly from Puppo et al. (2015) and Curto et al. (2025).

To evaluate computational performance, we compared running times of GBAS‐GUI with and without parallelization and recorded RAM usage to document resource requirements. Measurements for time and RAM usage were directly implemented as additional scripts using the python libraries time and psutil. To assess information content, we compared PIC values obtained from WAI‐ and AL‐based genotypes across all datasets. For the Spruce dataset, we additionally compared datasets obtained with and without paralog‐defining length intervals in potentially duplicated markers. Both matrices were compared in terms of number of markers kept after filtering according to a missing data threshold of 50%, as well as standard genetic diversity and population‐structure metrics. Genetic diversity was estimated by calculating average number of alleles, Shannon's information index, number of private alleles and expected heterozygosity per population. Genetic structure was quantified using pairwise *F*_ST_. Additionally, a principal coordinate analysis (PCoA) was used to evaluate population structure patterns based on genetic distance among individuals. Finally, an AMOVA analysis was conducted to test for genetic differentiation among populations in both datasets. All these analyses were performed using Genalex v6.5 (Peakall and Smouse 2006). Differences in PIC between WAI‐ and AL‐based genotypes across datasets and genetic diversity and structure parameters for Spruce datasets were evaluated using a paired Wilcoxon test as implemented in Base R (R Core Team 2025).

To assess sequence accuracy, we compared *Micromeria* consensus sequences produced by GBAS‐GUI with Sanger sequences generated by Puppo et al. (2014) and Puppo et al. (2015). Although Sanger sequencing has its own sources of error, these are independent of Illumina‐based biases, making concordant phylogenetic signal between the two methods an informative accuracy check. Phylogenetic trees for each marker were inferred using maximum likelihood with the RAxML‐NG online server (https://raxml‐ng.vital‐it.ch/#/; Kozlov et al. 2019). The best‐fitting substitution models were selected using jModelTest (Darriba et al. 2012).

## 3 Results

### 3.1 Performance Results

**FIGURE 6:**
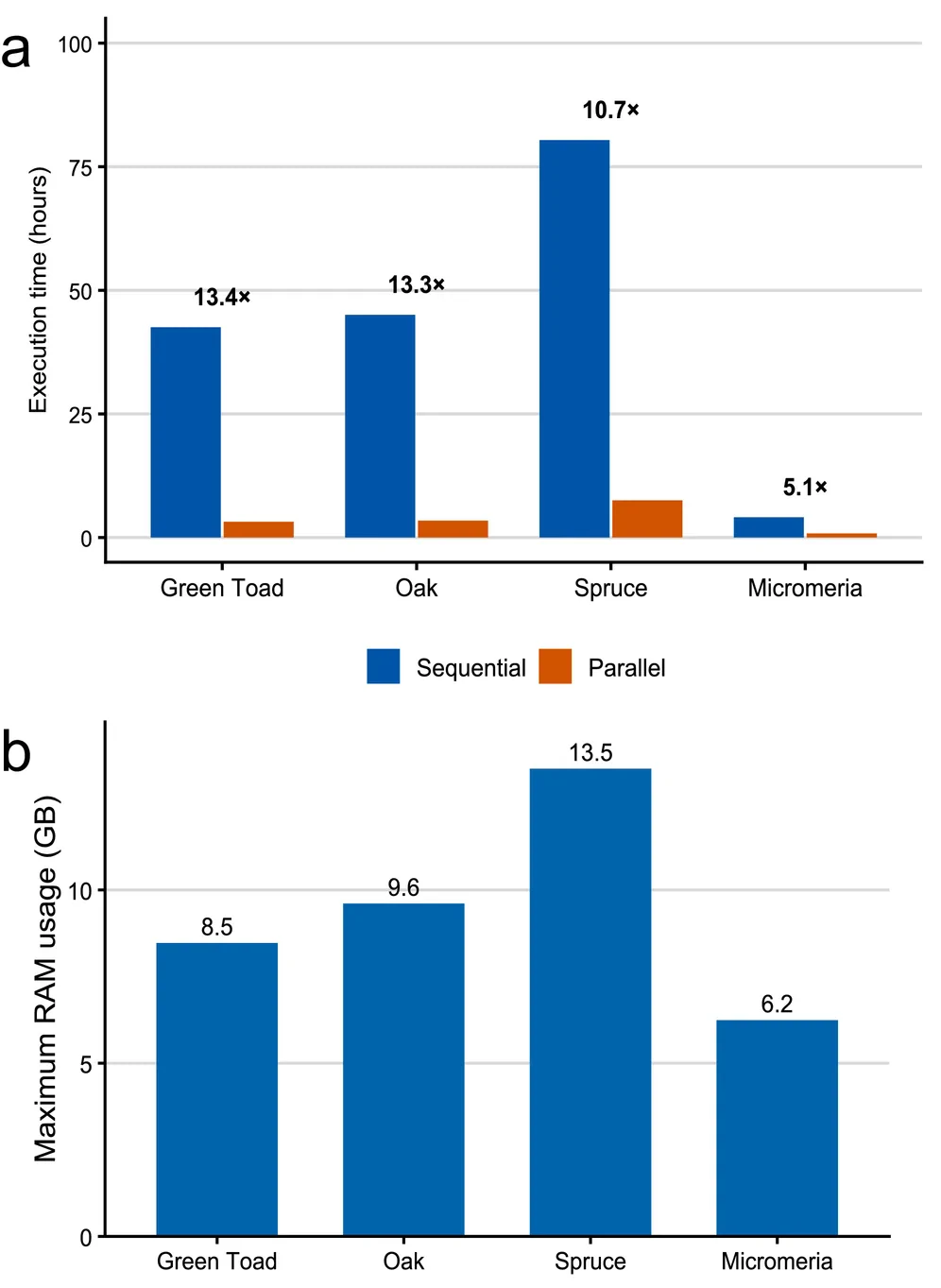
Performance comparison of GBAS‐GUI across four representative projects. (a) Comparison of execution time (hours) between sequential execution (single‐core) and parallel execution (multiple cores). Values above the bars indicate the speedup achieved through parallelization (sequential execution time divided by parallel execution time). (b) Maximum RAM usage recorded during pipeline execution for each project.

Performance efficiency is essential given the large number of reads processed by the pipeline, and the application of parallelization significantly reduces processing time. Figure 6 shows a speedup of between 5× to 13× when parallelization was used. Maximum RAM usage ranged from 6.4 GB to 13.8 GB, with higher memory requirements associated with an increased number of markers.

### 3.2 PIC Of AL and WAI

**FIGURE 7:**
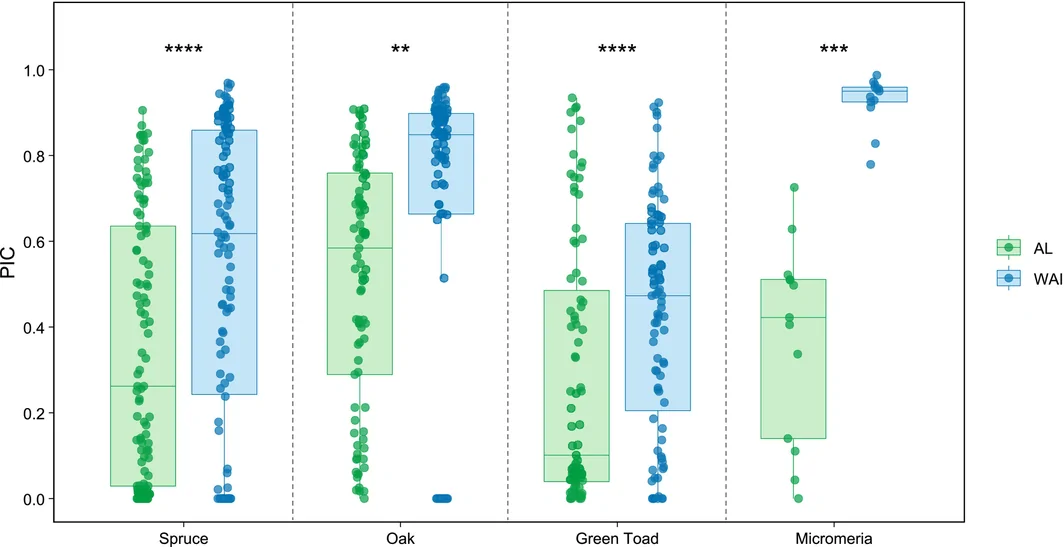
Boxplots per project showing PIC values per marker for genotyping based on amplicon length (AL) and whole amplicon information (WAI). Differences between AL and WAI PIC values were assessed using paired Wilcoxon signed‐rank tests. Asterisks indicate significance levels: *p < 0.05, **p < 0.01, ***p < 0.001 and ****p < 0.0001.

We used PIC values to quantify the gain in information content when using whole amplicon information compared to allele length. As expected, median PIC per marker increased for WAI genotypes compared with AL, showcasing the advantage of sequence‐based over length‐based genotyping (Figure 7). This trend was not observed across all markers. Depending on the project, AL had higher PIC values than WAI in up to 26% of polymorphic markers (Figure S1 and Table S3). These tended to have higher missing data in the WAI matrix than in AL.

### 3.3 Genotyping With and Without Rescuing Duplicated Loci

Out of the 147 markers used for the spruce dataset, 32 loci were identified as potentially duplicated after visual inspection of the amplicon length histogram plots. From these we were able to define between two and six amplicon length intervals per locus (Table S4). By rerunning the GBAS‐GUI each interval was considered as an independent locus, yielding 26 additional loci compared to running the pipeline without rescuing duplicated loci (Table S4). Two markers ended up being excluded after splitting according to potential paralogs since this process resulted in a reduction of read count, making either allele calling ambiguous or the total read count below the defined thresholds (Table S4).

Overall genetic diversity and structure parameters were slightly higher for the matrix including paralog correction than for the one without (Figure S2). However, this difference was just significant for expected heterozygosity. PCoA resulted in similar clustering patterns (Figure S3). By implementing this duplication rescue scheme, the Bergen population was slightly more separated from the remaining ones. Although the *F*_ST_ values obtained from the AMOVA were significant (Figure S3), they were low and remained virtually unchanged after implementing the duplication rescue approach (0.0390 and 0.0392, respectively).

### 3.4 Accuracy Results With Micromeria

For the three markers analyzed with GBAS, between 5 and 11 variable positions were found, from which 2 to 6 were informative. For Sanger variable positions ranged from 5 to 14, while the number of informative positions were the same. Further details plus information concerning the substitution models used are available in Table S5. *Micromeria* in the Canary Islands is characterized by two main lineages, one occupying the eastern islands of Lanzarote, Fuerteventura and Gran Canaria (Eastern lineage, Table S1) and the other occupying the western island of Tenerife, La Palma and El Hierro (Western lineage, Table S1) (Puppo et al. 2014; Puppo et al. 2015; Curto et al. 2018). Both lineages are found in the island of La Gomera (Puppo et al. 2014; Puppo et al. 2015; Curto et al. 2018; Table S1). All markers showed the split between these two lineages with high support values (Figure S4). Comparing both methods, ADK and SINAC1 showed no differences. For DXR, P428 groups with H25 in GBAS while with P477 in Sanger. The GBAS sequences from sample P428 were constructed from 165 reads and shows five differences compared to Sanger, all at heterozygous positions in GBAS that are homozygous in Sanger. The GBAS sequence from P477 resulted from a consensus of 47 reads and it differs in one position from Sanger (Table S6): a homozygous T in GBAS is a heterozygous T/C in Sanger. The GBAS sequence from sample H25 was a consensus of 407 reads and it is identical to Sanger. The samples H25 and P428 are both *M. helianthemifolia*, while P477 is *M. tenuis* , thus GBAS showed a phylogenetic signal congruent with species delimitation whereas Sanger did not.

## 4 Discussion

GBAS‐GUI has several key features that enhance the accessibility and robustness of GBAS analyses while broadening the pipeline's applicability to a wider range of biological questions and study systems. Below, we describe how each improvement contributes to this goal.

### 4.1 Graphical User Interface (GUI)

The GUI runs on both Unix and Windows systems and can be installed via *pip*, ensuring standardized configuration and minimizing environment‐dependent execution errors. By consolidating multiple processes, like quality control, demultiplexing and genotyping into one program, it simplifies usage and reduces the likelihood of user errors or misconfigured parameters. Dedicated menus for parameter selection and built‐in internal checks flag potential problems during or prior to execution. Each run also generates a configuration text file containing all parameters used, facilitating traceability, reproducibility and iterative optimization.

GBAS‐GUI allows users to re‐run specific modules of the pipeline independently, rather than restarting from the beginning. This significantly reduces computation time during parameter optimization and enables direct evaluation of how individual settings affect intermediate outputs. Together, these improvements make GBAS‐GUI more user‐friendly, reproducible and flexible across computational environments.

The GUI is also designed to facilitate quality control (QC), a key step in obtaining reliable genotype information given the heterogeneity typical of GBAS datasets. GBAS‐GUI tracks the number of sequences retained at each stage of analysis and stores intermediate data in accessible formats. Users can inspect amplicon length histograms and view allele sequences saved as FASTA files using standard visualization tools.

All intermediate files are preserved, allowing users to trace the analytical path of each locus and identify sources of data loss or error. For example, by inspecting marker plots and sequence alignments, we reveal loci derived from duplicated genomic regions in the spruce dataset. Furthermore, monitoring the number of reads retained across pipeline stages helps diagnose specific issues. Drops in read count in QC and demultiplexing indicate potential library‐preparation errors; in read merging, inaccurate predictions of expected amplicon size; in SNP identification or allele calling, the co‐amplification of nonspecific regions or poor locus performance. Such built‐in QC visualization and traceability tools empower users to identify problems early, optimize parameters and improve overall genotype reliability.

The applicability of GBAS extends well beyond academic research, reaching users who may not be familiar with command‐line bioinformatics. The GUI makes the analysis of GBAS data more user‐friendly, while the remaining features ensure stability, flexibility and traceability. As a result, GBAS‐GUI enables users outside specialized bioinformatics teams and potentially outside academia, to analyze GBAS data independently.

### 4.2 Relational Database

GBAS is particularly valuable for projects involving large sampling volumes, such as long‐term monitoring programs. These often require the integration of multiple sequencing runs originating from different locations, times or even species. For such projects, it is essential that genotypes remain comparable across runs and that associated metadata are compiled in a structured and accessible way. To address this need, we implemented a centralized relational database that grows dynamically as new data is added.

At its core, the database links each sequence to its corresponding genotype through the *allele_list* file. In that system, alleles were assigned numeric identifiers representing the final haplotype sequences detected in each run. Haplotypes from subsequent datasets are automatically compared against the existing *allele_list* to ensure that identical sequences across runs receive the same allele designation. Novel sequences are assigned to new allele identifiers and appended to the database.

All genotypic information is stored in a relational SQL database that integrates sample metadata. This allows genotype data from multiple runs and projects to be stored far more efficiently than with static file‐based lists.

In many research settings, projects may share samples, markers or both, and metadata can vary between studies. Managing such information across independent files is complicated and prone to inconsistency, especially when users need to extract specific subsets of the data. A relational database addresses this challenge by linking information that would otherwise reside in separate files, enabling fast and flexible queries. In GBAS‐GUI, users can retrieve genotypes based on marker names, sample metadata or combinations of both and can likewise extract metadata for specific genotype sets. Although the current implementation supports only three query fields, two of which are fixed (project and loci), the framework is readily expandable and can accommodate additional metadata types in future updates. This design, while conceptually simple, is essential for maintaining consistency, reproducibility and traceability in long‐term or multi‐site genotyping projects.

### 4.3 Improving Efficiency Through Multiprocessing

As discussed above, GBAS is particularly well suited for studies involving large sample sizes, but such datasets require efficient computational processing. By incorporating a multiprocessing architecture, we see that GBAS‐GUI achieves substantial improvements in processing speed compared to using a single processor. Parallelization is especially effective for components that operate on large numbers of independent files or sequences, such as demultiplexing and consensus‐sequence generation. These tasks scale almost linearly with the number of processing cores available, making them ideal candidates for multiprocessing. Despite these gains, further improvements in efficiency remain possible. For example, specific steps of the pipeline like demultiplexing could be accelerated through the integration of optimized third‐party tools.

### 4.4 Rescuing Orthologous Signal From Co‐Amplification Product

Eukaryotic genomes are characterized by extensive gene and genome duplication throughout their evolution, which can complicate the assessment of genetic variation among individuals (Koonin 2005). This results in multicopy gene families, pseudogenes and non‐coding regions duplicated across the genome. Marker development workflows typically attempt to filter out these loci (e.g., Chamala et al. 2015); however, these filters are often imperfect due to the highly repetitive nature of many genomes and the limited availability of high‐quality reference assemblies for non‐model species (Köbölkuti et al. 2020; Nystedt et al. 2013). Consequently, GBAS datasets may include markers that co‐amplify multiple genomic regions, making it difficult to retrieve orthologous genotypes. This ambiguity can be further exacerbated by amplification artefacts and the sequencing of nonspecific products. Such loci are usually excluded after genotyping, yet if orthologous signals can be rescued, they can still provide valuable information that would otherwise be lost.

Many co‐amplified products exhibit fixed insertions or deletions, resulting in distinct ranges of amplicon lengths. The GBAS‐GUI pipeline takes advantage of this property by defining expected length windows for each putative copy while excluding ranges corresponding to nonspecific amplification products. Genotypes are then called separately within these windows, effectively treating each copy as an independent marker. This approach allows the recovery of genotype information from co‐amplification products, minimizing the loss of data associated with their exclusion.

In the spruce dataset, for instance, we recovered genotype information for an additional 26 markers identified as potentially duplicated loci. Although the large number of loci in this dataset meant that the inclusion of rescued duplicates had only a small effect on the overall results, this feature can have a substantial impact in studies with fewer markers, where each locus contributes more strongly to downstream analyses.

Despite its usefulness, this approach has certain limitations. First, genotyping intervals must be identified prior to analysis. This is currently achieved by visual inspection of amplicon length histograms to detect co‐amplification patterns indicative of gene duplication or unspecific product. While feasible for datasets with few markers, this becomes time‐consuming in large‐scale studies. Therefore, automated detection methods should be developed in future versions. Second, if the amplicon length ranges of multiple copies or unspecific products, the current approach cannot reliably separate their signals. Additional strategies, such as clustering based on sequence similarity (Ortiz et al. 2026) or inference of phylogenetic relationships among haplotypes (Johnson et al. 2016; Zhou et al. 2022), could help resolve such cases and may be integrated into future versions of the pipeline.

### 4.5 Added Value of Incorporating Sequence Information

GBAS‐GUI follows a stepwise strategy to reduce data complexity and sequencing noise by first defining candidate amplicon‐length genotypes consistent with the expectation of diploid organisms and subsequently identifying potential heterozygous SNP sites used to reconstruct haplotypes and determine final allele calls. This approach allows efficient genotype calling by incorporating all sequence variations captured within an amplicon. As a result, it increases the information content of markers traditionally genotyped using amplicon length alone, such as microsatellites.

Incorporating sequence variation generally increases the number of detected alleles and, consequently, the polymorphism information content (PIC), which was observed for most markers across the example datasets. However, a subset of markers showed the opposite pattern, with lower PIC values when genotypes were defined using whole amplicon information (WAI) compared to amplicon length (AL). Closer inspection revealed that these markers contained a higher proportion of missing data in the WAI‐based genotype matrices.

In the SSR‐GBAS pipeline, WAI‐based genotypes are assigned only when sufficient sequencing depth is available to unambiguously resolve haplotypes. Otherwise, genotypes are recorded as missing. Consequently, when sequence variation subdivides read depth among multiple haplotypes, some may fail to meet the requirements for confident assignment, leading to a reduction in observed variation and PIC. This situation can arise from inefficient amplification of a given marker, resulting in low overall sequencing depth or from high apparent sequence diversity caused by co‐amplification of non‐homologous regions or PCR artefacts such as chimaeras. In such cases, relying solely on length‐based genotypes is also inadvisable. In the former scenario, length alleles are likely homoplasic because additional SNP variation exists within length classes, whereas in the latter scenario, length variation may reflect amplification artefacts rather than true allelic differences. Thus, a decrease in PIC associated with WAI‐based genotyping serves as an indicator of problematic markers, either due to insufficient sequencing depth for reliable genotype calling or the presence of underlying artefacts. Comparing PIC values derived from WAI and AL genotypes therefore provides a useful diagnostic criterion for identifying markers that require further scrutiny or exclusion.

Obtained sequence information alongside genotype information means a wider applicability in terms of analytical frameworks. Genotypic data can be employed in frequency‐based population genetic analyses, such as estimating genetic diversity and population structure. In parallel, the sequence data can support analyses based on haplotypes, including the construction of haplotype networks, the estimation of nucleotide diversity and tests of neutrality such as Tajima's D. The combination of both data types also enables the use of GBAS data in phylogenetic and phylogeographic contexts. Other studies have already taken advantage of this property. For example, Curto et al. (2025) analyzed variation of *Micromeria* species across the Canary Islands using both a phylogenetic and population genetics framework using the same EPIC and chloroplast markers.

### 4.6 Comparison With Existing Pipelines

Although several pipelines and analytical frameworks are available for generating genotype information from GBAS data, to our knowledge none integrates all the key features implemented in the current version of GBAS‐GUI, namely the use of complete sequence information for genotype inference, visual inspection tools, a graphical user interface, a built‐in relational database and the ability to recover information from duplicated loci. These differences also complicate direct benchmarking because existing pipelines often differ substantially in their analytical scope, including the types of genetic variation considered (e.g., length polymorphisms, SNPs or haplotypes), the marker systems they support (e.g., requiring the presence of tandem‐repeat motifs) and the stage of the analytical workflow from which they operate. Consequently, comparisons of execution time or computational performance would not reflect equivalent analytical tasks and would therefore provide limited insight into pipeline performance. For this reason, we compare GBAS‐GUI with existing pipelines qualitatively by summarizing their main characteristics in Table 1. Nevertheless, the lack of direct benchmarking limits our ability to quantitatively assess the relative strengths and limitations of GBAS‐GUI compared with alternative approaches. Future development of comparable GBAS tools and standardized benchmark datasets should enable more comprehensive evaluation of alternative analytical strategies and facilitate further improvements in GBAS data analysis.

**TABLE 1:** Comparison among existing pipelines able to analyze GBAS data.

Program	Author	Year	Genotyping strategy	Genotype information				Requirements			Output files	Important features					
				SSR	Other indels	SNP	WAI	SSR containing seq.	Reference seq.	Input files		Automatic genotyping	Deals with paralogues	Manual control	GUI	Database	Op. System
*GBAS‐GUI*	*This study*		*Variant depth ratios and filtering*	*Y*	*Y*	*Y*	*Y*	*N*	*N*	*fastq, metadata*	*Genotype table, genotype plots, sequence and database*	Y	Y	*Y*	*Y*	*Y*	*All*
SSR‐GBAS	Curto et al.	2019	Variant depth ratios and filtering	Y	Y	Y	Y	Y	N	fastq, metadata	Genotype table, genotype plots and sequence	Y	N	Y	N	N	All
AMGT‐TS	Huo et al.	2021	Filtering low frequency variants	Y	N	N	N	Y	Y	fastq/a, reference and metadata	Genotype table, genotype plots, sequence alignments and report	Y	N	N	N	N	Unix
AMPLISAS	Sebastian et al.	2016	Clustering and filtering	N	Y	Y	Y	N	N	fastq/a and metadata	Genotype table, sequences alignments	Y	Y	N	Web	N	All, Web
AmpSeq‐SSR	Li et al.	2017	Variant depth ratios	Y	N	N	N	Y	Y	fastq/a, reference and metadata	Genotype table, allele file	Y	N	N	N	N	Unix
CHIIMP	Barbian et al.	2018	Filtering low frequency variants	Y	Y	Y	Y	Y	N	Demultiplexed fastq/a and metadata	Genotype table, genotype plots, sequence alignments and report	Y	N	Y	N	N	All
FDSTools	Hoogenboom et al.	2017	Stutter and or allele modelling	Y	N	Y	N	Y	N	Merged and demultiplexed fasta	Genotype table, report	Y	N	Y	N	Y	All
GTseq‐Pipeline	Campbell et al.	2015	Variant depth ratios	N	N	Y	Y	N	N	fastq/a and metadata	Genotype table	Y	N	N	N	N	Unix
HaplotypR	Lerch et al.	2017	Clustering and filtering	N	N	Y	N	N	Y	fastq	Haplotype list	Y	N	N	N	N	All
HipSTR	Willems et al.	2017	Stutter and or allele modelling	Y	Y	Y	Y	Y	Y	BAM files, reference, metadata	vcf file	Y	N	N	N	N	Unix
jMHC	Stuglik et al.	2011	Visual inspection of most abundant variants	N	N	Y	Y	N	N	fastq/a and metadata	Alignment files	N	Y	Y	N	N	All
MEGASAT	Zhan et al.	2017	Variant depth ratios	Y	Y	N	N	N	N	fastq/a and metadata	Genotype table, genotype plots	Y	N	Y	Y	N	All
MicNeSs	Suez et al.	2016	Stutter and or allele modelling	Y	N	N	N	Y	N	Merged and demultiplexed fasta	Genotype table	Y	N	N	N	N	All
NGS‐μsat	Roy et al.	2021	Stutter and or allele modelling	Y	N	N	N	Y	N	fastq/a and metadata	Genotype table, genotype plots	Y	N	Y	N	N	All
RepeatSeq	Highnam	2012	Stutter and or allele modelling	Y	N	N	N	Y	Y	BAM files, reference, metadata	Genotype table	Y	N	N	N	N	Unix
Seq2Sat	Liu et al.	2023	Variant depth ratios	Y	Y	Y	N	Y	N	fastq/a and metadata	Genotype table, genotype plots, sequence alignments and report	Y	N	Y	Web	N	All, Web
SESAME	Meglécz et al.	2011	Visual inspection of most abundant variants	N	Y	Y	Y	N	N	fastq/a and metadata	Haplotype list	N	N	Y	Y	N	All
SSRSeq	Cui et al.	2020	Stutter and or allele modelling	Y	Y	N	N	N	Y	fastq/a, reference	Genotype table	Y	Y	Y	N	N	All
SSR‐seq	Šarhanová et al.	2018	Visual inspection of most abundant variants	Y	Y	Y	Y	N	N	fastq/a and metadata	Haplotype list	N	N	Y	N	N	Unix
STRaitRazor	King et al.	2017	Filtering low frequency variants	Y	Y	Y	Y	Y	N	fastq/a, database	Genotype table	Y	N	Y	Y	Y	Unix, Web
Target SSR‐Seq	Yang et al.	2019	Variant depth ratios	Y	N	N	N	Y	Y	fastq/a, reference and metadata	Genotype table	Y	N	N	N	N	Unix
toaSTR	Ganschow et al.	2018	Stutter and or allele modelling	Y	N	N	N	Y	N	fastq/a and metadata	Genotype table	Y	N	N	Web	N	Web

Abbreviations: N, No; Y, Yes.

Most existing tools are primarily designed for microsatellite genotyping. As a result, they often include specialized filters, such as the detection of tandem repeat motifs, that make their implementation incompatible with other marker systems like nuclear genes (e.g., Ganschow et al. 2018; Hoogenboom et al. 2017; King et al. 2017; Yang et al. 2019). Among pipelines that are not strictly SSR‐oriented, many still focus on a specific variant type through their allele‐calling or filtering strategies. For example, GTseq‐Pipeline (Campbell et al. 2015) and jMHC (Stuglik et al. 2011) target SNP and haplotype variation, respectively and apply filtering schemes that may overlook complex length polymorphisms. Conversely, several other programs consider only length variation for allele calling and do not integrate full amplicon sequence information. Even tools that detect both SNPs and indels, such as Seq2Sat (Liu et al. 2024), typically treat them independently rather than merging them into unified allele definitions.

In terms of genotype inference, the only program producing outputs comparable to GBAS‐GUI under similar analytical requirements is Chiimp (Barbian et al. 2018). Chiimp performs gradual filtering of potential alleles based primarily on sequence depth. In contrast, GBAS‐GUI separates variation sources by first resolving allele length and read‐depth ratios, and only then evaluating SNP‐defined haplotypes. This staged approach is particularly suited to markers where microsatellite repeat variation and SNP variation co‐occur. Although we did not perform a direct comparison, this separation of variation types may help reduce data complexity in such loci.

Visualization is another key component of genotype validation. While most pipelines provide graphical output, only a few allow users to manually adjust allele‐length selection during genotyping. Seq2Sat (Liu et al. 2024) offers an interactive graphical interface that enables users to modify length selections and thereby influences downstream haplotype resolution In GBAS‐GUI, such modifications are technically possible by editing intermediate files, but this approach goes beyond the program's rationale of efficiently analyzing large marker sets. In practice, users are therefore encouraged to exclude problematic markers rather than manually adjust allele‐length selections. Nevertheless, implementing an interactive interface similar to that of Seq2Sat could be useful for other purposes, such as defining amplicon‐length intervals to rescue orthologous signals from co‐amplified loci.

More generally, graphical interfaces remain uncommon among GBAS pipelines, and when available, they are often web‐based. While web interfaces facilitate remote accessibility, the offline GUI of GBAS‐GUI provides a key advantage for running analyses independently of internet connectivity and external servers.

A further distinctive feature of GBAS‐GUI is its ability to build and manage a project‐specific allele/haplotype database that includes associated metadata (e.g., sample identifiers, experimental parameters, population information or ecological context). Among the tools we surveyed, STRaitRazor2 (King et al. 2017) and FDSTools (Hoogenboom et al. 2017) also incorporate database functionality. STRaitRazor maintains a curated reference database of approximately 2500 known STR alleles, and FDSTools can generate reference databases for stutter and noise modelling. However, these are predefined and tailored to specific applications, and we found no evidence that they allow management or embedding of user‐defined metadata to the same extent as GBAS‐GUI.

Finally, GBAS‐GUI is one of the few pipelines capable of retaining and interpreting information from duplicated genomic regions. SSRseq (Cui et al. 2022) was developed for polyploid organisms and effectively handles SSR variation but does not extend to more complex, multi‐variant sequence markers. Conversely, AMPLISAS (Sebastian et al. 2016) and jMHC (Stuglik et al. 2011) are optimized for multi‐copy gene families but are not designed for large‐scale genotyping of orthologous loci across multiple samples. GBAS‐GUI combines the ability to distinguish duplicated alleles with efficient scalability across loci and datasets, expanding its applicability to a wider range of biological systems.

GBAS‐GUI is currently implemented within a diploid framework and is therefore not completely applicable to polyploid organisms. The genotyping amplicon length intervals can produce diploid genotypes out of orthologous genes from allopolyploids originating from different species. However, the same cannot be applied to autopolyploid organisms. Given the high prevalence of polyploidy in many taxa (Wood et al. 2009), particularly plants, future versions of the pipeline should consider extending the allele‐calling algorithm to accommodate genotypes of higher ploidy levels. The allele dosage correction approach described by Cui et al. (2022), which infers allele dosage by comparing observed read counts with expectations under a specified ploidy level, could provide a useful starting point for such developments.

GBAS‐GUI builds largely upon the SSR‐GBAS pipeline (Curto et al. 2019), retaining the same fundamental allele‐calling strategy. However, GBAS‐GUI substantially extends the functionality and usability of SSR‐GBAS (Table 1). SSR‐GBAS consists of a collection of standalone Python and R scripts that must be run sequentially, making the workflow more complex and requiring familiarity with command‐line and script‐based analyses. In addition, SSR‐GBAS is restricted to SSR markers and requires the presence of a repeat motif within the amplified region, does not support parallel computing or integrated database management and does not implement the rescue of potentially duplicated loci. Consequently, when applied to SSR loci using a single core and without duplicated‐locus rescue, GBAS‐GUI is expected to produce equivalent genotyping results to SSR‐GBAS with similar running times, while providing a substantially more integrated and user‐friendly workflow and more efficient data management through its relational database. Thus, GBAS‐GUI incorporates all the functionality of SSR‐GBAS while extending its applicability, computational efficiency and analytical capabilities.

## 5 Conclusions and Future Directions

We present GBAS‐GUI, a flexible and broadly applicable tool across diverse, non‐model study systems and research questions. By integrating a graphical user interface, it simplifies and increases the robustness of GBAS data analysis. In addition, it introduces several key innovations, including the implementation of a relational database, the recovery of genotype information from duplicated loci and the ability to process marker systems beyond microsatellites. Together, these features substantially broaden the analytical scope of genotyping‐by‐amplicon sequencing (GBAS).

The main objective of these developments was to ensure that GBAS remains feasible for projects involving large sampling volumes while remaining accessible to non‐specialists. This is particularly relevant for practitioners in fields such as biodiversity conservation who wish to assess the outcomes of management actions through genetic monitoring. Moreover, the capacity to genotype large sampling sizes across different marker types may unravel new patterns of genetic diversity that might be overlooked by conventional approaches.

Future versions of GBAS‐GUI will focus on increasing interactivity and automation. Enhancing interactivity of visualization tools, such as real‐time adjustment of amplicon‐length histograms, will further streamline quality control. Automated detection of duplicated loci and improved discrimination among paralogs with overlapping amplicon lengths will expand applicability to highly duplicated genomes. Finally, extending the range of metadata fields will enable users to tailor the database to specific research or monitoring projects. Together, these developments will build on what GBAS‐GUI has already established: a more standardized, reproducible and accessible framework for GBAS data generation and analysis.

## Author Contributions

M.C., S.S. and H.M. contributed to the conceptualization and experimental design of the study. Field work and laboratory work were conducted by C.R., T.V., Y.P.K, M.G. and G.K. Sequence data was analyzed by S.S., while statistical analysis was performed by S.S. and M.C. C.R., H.D. and T.V. contributed throughout the analysis's steps. M.C. and S.S. wrote the first draft of the manuscript that was revised and edited by the remaining authors. All authors contributed to the article and approved the submitted version.

## Funding

Work on the design of GBAS‐GUI was funded by the project “ATIV Biodat” granted within the call “(Digitale) Forschungsinfrastruktur” by BMFWF, Austria, and funded by the European Union – NextGenerationEU. Sampling and genotyping was mostly funded by the Bundesministerium für Landwirtschaft, Regionen und Tourismus (references 101660, and GZ BMLRT 2021‐0.370.496).

## Disclosure

Benefit‐Sharing Statement: There are no benefits to report.

## Ethics Statement

This study did not involve human participants. Plant material consisted of leaf samples, and animal genetic material was obtained using non‐invasive buccal swabs from green toads ( *Bufotes viridis* ). Animals were handled only for the time required to collect samples, following procedures designed to minimize stress and avoid harm and were released immediately after sampling. All sampling was conducted under the appropriate permits issued by the relevant authorities.

## Conflicts of Interest

The authors declare no conflicts of interest.

## Supporting information


**Figure S1:** Comparison per marker of PIC values calculated for genotyping based on allele length (AL) and whole amplicon information (WAI).
**Figure S2:** Genetic diversity and differentiation measures comparison between spruce datasets with (Pos) and without duplication control (Pre). Asterisks indicate significant differences based on Wilcoxon tests (*p* < 0.05, *p* < 0.01, *p* < 0.001, *p* < 0.0001; ns, not significant).
**Figure S3:** Principal coordinate analysis among individuals for the spruce dataset with and without duplication control. AMOVA was used to test for genetic differentiation among populations, with the corresponding FST and p‐values reported in each plot.
**Figure S4:** Comparison of Phylogenetic analysis of EPIC marker sequences produced with Illumina (right) and Sanger (left). Clades covered by the blue boxes correspond to the Western lineage while the ones by the orange boxes to the Eastern lineages.

**Table S1:** Sample information per dataset.
**Table S2:** List of markers used per dataset.
**Table S3:** Number of polymorphic markers with higher PIC based on allele length (PIC_AL > PIC_WAI) or whole‐amplicon information (PIC_WAI > PIC_AL).
**Table S4:** Spruce markers identified as potentially duplicated, indicating the percentage of individuals containing genotypes. Values above 50% are marked in green. For the dataset implementing duplication control, these values are separated by paralog. Each paralog was treated as an independent locus.
**Table S5:** Variable and informative positions for Micromeria alignments produced with Sanger or Illumina technologies. Additionally, substitution models used for phylogenetic analysis are presented.
**Table S6:** Positions differing between GBAS and Sanger sequences for the Micromeria project. Results are shown per marker, sample and alignment position. The type of difference in the GBAS consensus sequence (substitution, deletion or insertion) is indicated, together with the corresponding genotype. Additional information on read depth and amplicon length for GBAS is also provided.

## Data Availability

Raw sequencing data for the Oak, Green Toad and Spruce datasets are available at the SRA repository from GenBank associated with the bioproject PRJNA1445872, while for *Micromeria* with the bioproject PRJNA1194140. All code from the GBAS‐GUI is available and described at https://github.com/sonnenbe‐dot/GBAS‐GUI.
