# Supplementary material for: Upscaling Genotyping by Amplicon Sequencing With GBAS‐GUI
Source: Mol Ecol Resour. 2026 Sep 4;26(7):e70198. doi: 10.1111/1755-0998.70198 (PMC13544635; doi:10.1111/1755-0998.70198)
Supplement: Supplementary file 1 [file MEN-26-e70198-s002.docx]

**Supplementary Information:**

**Table of contents:**

| Figure S1. - Comparison per marker of PIC values calculated for genotyping based on allele length (AL) and whole amplicon information (WAI). | Page 2 |
| --- | --- |
| Figure S2. Genetic diversity and differentiation measures comparison between spruce datasets with (Pos) and without duplication control (Pre). Asterisks indicate significant differences based on Wilcoxon tests (P < 0.05, P < 0.01, P < 0.001, P < 0.0001; ns, not significant). | Page 3 |
| Figure S3. Figure S3. Principal coordinate analysis among individuals for the spruce dataset with and without duplication control. AMOVA was used to test for genetic differentiation among populations, with the corresponding $F_{ST}$and *P*-values reported in each plot. | Page 4 |
| Figure S4. Comparison of Phylogenetic analysis of EPIC marker sequences produced with Illumina (right) and Sanger (left). Clades covered by the blue boxes correspond to the Western lineage while the ones by the orange boxes to the Eastern lineages. | Page 5 |

Figure S1. Comparison per marker of PIC values calculated for genotyping based on allele length (AL) and whole amplicon information (WAI).

Figure S2. Genetic diversity and differentiation measures comparison between spruce datasets with (Pos) and without duplication control (Pre). Asterisks indicate significant differences based on Wilcoxon tests (P < 0.05, P < 0.01, P < 0.001, P < 0.0001; ns, not significant).

Without duplication control

Coord. 2

| Coord. 1 | Bergen |
| --- | --- |
| 0.187599 | 0.679981 |
| -0.0313397 | -0.0432479 |
| -0.389525 | 0.457138 |
| -0.13037 | 0.848896 |
| 0.886847 | 0.769308 |
| 1.03935 | 0.860574 |
| -0.0451458 | 0.342018 |
| -0.177095 | 0.28042 |
| 1.8236 | 0.752338 |
| 0.441399 | 0.696567 |
| -0.0910963 | 0.895043 |
| -0.382335 | 0.185384 |
| 0.60038 | 0.674224 |
| -0.179364 | 0.675339 |
| -0.0427572 | 0.745063 |

| Coord. 1 | Flurwald |
| --- | --- |
| 1.02802 | -0.659161 |
| -0.558187 | -0.278467 |
| 0.796549 | -1.12266 |
| 0.222756 | -0.578556 |
| 1.11974 | -1.19634 |
| -0.740961 | -0.139657 |
| -1.12116 | -0.00370875 |
| -0.492656 | -0.266027 |

| Coord. 1 | Muhr |
| --- | --- |
| 0.862987 | 0.0621029 |
| -1.12113 | -0.290917 |
| 0.506755 | 0.164736 |
| -0.788413 | -0.507302 |
| 0.754301 | 0.359128 |
| 0.373011 | 0.0757082 |
| 1.01655 | -1.02724 |
| 0.208535 | 0.288909 |
| 0.556401 | 0.0336863 |
| -0.860195 | -0.305593 |
| -0.836179 | -0.180371 |
| -0.820963 | -0.121884 |
| 0.991449 | 0.620621 |

| Coord. 1 | Niedersachsen |
| --- | --- |
| 1.14028 | -1.1661 |
| 1.78394 | -0.502808 |
| -0.498314 | -0.260594 |
| 0.0585716 | -0.100696 |
| -0.629755 | -0.402519 |
| 1.44124 | -1.39336 |
| -0.475932 | -0.736139 |
| -0.774986 | -0.309286 |
| -0.886152 | 0.10831 |
| -1.0479 | -0.29081 |
| 0.322656 | -0.214952 |
| 0.409096 | 0.110131 |
| -0.0654641 | -0.146408 |

| Coord. 1 | Rothwald |
| --- | --- |
| -0.366169 | 0.363123 |
| 0.0641739 | 0.527982 |
| -0.29419 | 0.0653823 |
| -0.273919 | 0.337484 |
| 0.0536818 | 0.210614 |
| -0.174692 | 0.350711 |
| -0.12161 | 1.08514 |
| -0.471568 | 0.735437 |
| -0.499443 | -0.373076 |
| -0.430112 | 0.467099 |
| -0.522931 | 0.511954 |
| -0.588183 | -0.204238 |
| -0.669473 | 0.272952 |
| -0.730521 | -0.0188207 |
| -0.228287 | 1.09483 |
| 0.915774 | 0.967824 |
| -0.694658 | 0.25222 |

| Coord. 1 | Semslach |
| --- | --- |
| -0.803932 | -0.395132 |
| 0.63169 | 0.577745 |
| -0.549676 | 0.219784 |
| -1.14795 | -0.201088 |
| -0.639471 | -0.665128 |
| 0.686188 | -1.00277 |
| -0.418372 | -0.321688 |
| 0.0678725 | 0.313048 |
| 0.331376 | -0.23345 |
| 0.800986 | 0.472723 |
| 0.125365 | -0.477036 |
| 0.382208 | 0.267783 |
| -0.553445 | -0.38361 |
| 0.562222 | -0.0881702 |
| -0.378869 | -0.344099 |

| Coord. 1 | Silbertal |
| --- | --- |
| -0.599207 | 0.15332 |
| -0.688026 | -0.186291 |
| 0.92476 | -0.373831 |
| 0.0359906 | -0.00481531 |
| -0.395999 | -0.539308 |
| 0.530592 | 0.267089 |
| 0.793836 | 0.565528 |
| -0.70833 | -0.299679 |
| 0.44585 | -0.443876 |

| Coord. 1 | Weinsbergerwald |
| --- | --- |
| -0.0122661 | -0.559731 |
| -0.911253 | -0.207401 |
| -0.715549 | 0.217145 |
| 0.176438 | -0.216517 |
| 1.29669 | 0.231331 |
| 0.561533 | 0.0204045 |
| 0.672992 | -0.101418 |
| 0.126149 | -0.679432 |
| -0.687768 | -0.20974 |
| 0.744372 | -0.415208 |
| -1.03951 | -0.0439189 |

AMOVA: FST=0.0392, P = 0.000

With duplication control

Coord. 2

| Coord. 1 | Bergen |
| --- | --- |
| 0.0570205 | 0.819069 |
| 0.192516 | 0.011979 |
| 0.436063 | 0.588452 |
| 0.350035 | 0.999642 |
| -1.02503 | 0.79695 |
| -1.08544 | 0.953922 |
| 0.105394 | 0.742192 |
| 0.283693 | 0.0832648 |
| -1.9827 | 1.1975 |
| -0.250328 | 1.12996 |
| 0.318714 | 1.16609 |
| 0.509113 | 0.359823 |
| -0.401458 | 1.1736 |
| 0.411019 | 0.964224 |
| 0.138709 | 1.00186 |

| Coord. 1 | Flurwald |
| --- | --- |
| -1.034 | -0.325749 |
| 0.569761 | -0.226595 |
| -1.06367 | -1.07144 |
| -0.460438 | -0.595801 |
| -1.25767 | -0.786033 |
| 0.707746 | -0.160159 |
| 1.12551 | -0.143613 |
| 0.464757 | -0.526648 |

| Coord. 1 | Muhr |
| --- | --- |
| -1.06972 | -0.0111397 |
| 1.15937 | -0.327567 |
| -0.356073 | 0.21046 |
| 0.94441 | -0.231926 |
| -0.875457 | 0.234628 |
| -0.562928 | 0.0311021 |
| -1.24763 | -0.816293 |
| -0.132755 | 0.227084 |
| -0.708971 | -0.127184 |
| 1.00028 | -0.0599529 |
| 0.93369 | -0.19375 |
| 0.864751 | -0.143222 |
| -1.05391 | 0.466731 |

| Coord. 1 | Niedersachsen |
| --- | --- |
| -1.34484 | -0.495885 |
| -1.86094 | -0.0874229 |
| 0.655802 | -0.131355 |
| -0.208681 | -0.602402 |
| 0.684573 | -0.349556 |
| -1.52243 | -0.835648 |
| 0.546884 | -0.473989 |
| 0.844334 | -0.437739 |
| 0.987663 | 0.0744554 |
| 1.06609 | -0.604804 |
| -0.427987 | -0.51187 |
| -0.242923 | 0.131957 |
| -0.104486 | -0.361999 |

| Coord. 1 | Rothwald |
| --- | --- |
| 0.409645 | 0.399813 |
| -0.178633 | 0.30163 |
| 0.34286 | 0.232204 |
| 0.419961 | 0.492967 |
| -0.191608 | 0.151032 |
| 0.346658 | 0.466001 |
| 0.337441 | 1.22133 |
| 0.599554 | 0.855179 |
| 0.561659 | -0.233149 |
| 0.494397 | 0.545771 |
| 0.579932 | 0.731196 |
| 0.625996 | 0.0367896 |
| 0.670726 | 0.244231 |
| 0.726733 | -0.105258 |
| 0.40015 | 1.11638 |
| -0.655776 | 1.26259 |
| 0.864389 | 0.28581 |

| Coord. 1 | Semslach |
| --- | --- |
| 0.795215 | -0.517603 |
| -0.800337 | 0.293665 |
| 0.707941 | 0.0930383 |
| 1.17972 | -0.229348 |
| 0.703783 | -0.725111 |
| -0.789433 | -0.822419 |
| 0.450413 | -0.368211 |
| 0.114958 | 0.292932 |
| -0.49598 | -0.443523 |
| -0.982961 | 0.0531078 |
| -0.299517 | -0.906398 |
| -0.627503 | 0.12795 |
| 0.57997 | -0.905473 |
| -0.844674 | -0.331327 |
| 0.483515 | -0.439101 |

| Coord. 1 | Silbertal |
| --- | --- |
| 0.686899 | 0.0748219 |
| 0.737726 | -0.226793 |
| -1.0634 | -0.538047 |
| 0.0131707 | -0.229414 |
| 0.45019 | -0.573642 |
| -0.676554 | 0.255734 |
| -0.927468 | 0.27247 |
| 0.840232 | -0.223271 |
| -0.675648 | -0.684112 |

| Coord. 1 | Weinsbergerwald |
| --- | --- |
| 0.0898045 | -0.577453 |
| 1.00041 | -0.507946 |
| 0.751884 | -0.178361 |
| -0.0751451 | -0.431872 |
| -1.35028 | 0.305529 |
| -0.833288 | -0.18443 |
| -0.865596 | -0.374846 |
| -0.393538 | -0.883079 |
| 0.612827 | -0.446852 |
| -0.975063 | -0.490955 |
| 1.04619 | -0.259393 |

Figure S3. Principal coordinate analysis among individuals for the spruce dataset with and without duplication control. AMOVA was used to test for genetic differentiation among populations, with the corresponding $F_{ST}$and *P*-values reported in each plot.

Figure S4. Comparison of Phylogenetic analysis of EPIC marker sequences produced with Illumina (right) and Sanger (left). Clades covered by the blue boxes correspond to the Western lineage while the ones by the orange boxes to the Eastern lineages.
